# Isotretinoin Treatment for Autosomal Recessive Congenital Ichthyosis in a Golden Retriever

**DOI:** 10.3390/vetsci9030097

**Published:** 2022-02-22

**Authors:** Ana Petak, Ivan-Conrado Šoštarić-Zuckermann, Marko Hohšteter, Nikša Lemo

**Affiliations:** 1 Clinic for Small Animals “Buba”, Dore Pfanove 11, 10 000 Zagreb, Croatia; 2 Department of Veterinary Pathology, Faculty of Veterinary Medicine University of Zagreb, Heinzelova 55, 10 000 Zagreb, Croatia; isostaric@vef.hr (I.-C.Š.-Z.); marko.hohsteter@vef.hr (M.H.); 3 Clinic for Internal Diseases, Faculty of Veterinary Medicine University of Zagreb, Heinzelova 55, 10 000 Zagreb, Croatia; nlemo@vef.hr

**Keywords:** ichthyosis, isotretinoin, *PNPLA 1*, golden retriever, autosomal recessive congenital ichthyosis

## Abstract

Ichthyoses are hereditary cornification disorders that manifest with abnormal differentiation and desquamation of keratinocytes in a form of generalized dry and scaly skin. In golden retriever dogs, autosomal recessive congenital ichthyosis (ARCI) has been associated with mutations in the *PNPLA 1* gene. In human medicine, isotretinoin is frequently used to treat ARCIs. The aim of this study was to investigate the clinical and histological effects of isotretinoin on ARCI in a golden retriever dog with confirmed mutation in the *PNPLA 1* gene. Clinical examination, blood analysis and histopathological examinations were conducted before and after 90 days of isotretinoin therapy. The clinical and histopathological findings indicate that treatment with oral isotretinoin was effective in improving ichthyosis without any side-effects.

## 1. Introduction

Ichthyoses represent a large heterogenous group of hereditary cornification disorders that manifest with abnormal differentiation and desquamation of keratinocytes in a form of generalized dry and scaly skin, variable erythroderma and hyperkeratosis [1,2]. Autosomal recessive congenital ichthyosis (ARCI) has been associated with mutations in the patatin-like phospholipase domain-containing protein 1 (*PNPLA 1*) gene in dogs and humans [3,4]. The *PNPLA 1* gene encodes enzymes that are important in lipid metabolism [5]. In human medicine, retinoids are frequently used for treatment of ichthyoses because of their ability to modulate epidermal proliferation, differentiation and inflammation [6,7].

Current therapy recommendations for canine ichthyoses are focused on removing the scales and improving the epidermal barrier with topical keratolytic, keratoplastic and/or moisturizing products [8], together with oral fatty acid supplementation [9], but unfortunately with variable improvements.

This case report describes clinical and histological improvement following isotretinoin treatment in a golden retriever dog with ARCI.

## 2. Materials and Methods

This investigation was conducted at the Veterinary Teaching Hospital (VTH) of the Faculty of Veterinary Medicine at the University of Zagreb with written owner consent.

### 2.1. Dermatological Examination

At day 0, a dermatologist examined the patient to assess the severity of scaling, the size and distribution of scales, and the presence and intensity of pruritus. An eight-year-old spayed female golden retriever was admitted to the VTH due to severe pruritus and severe scaling of the skin that was present from a very young age. Axillar and inguinal hyperpigmentation were present. Based on dermatological examination, differential conditions included ichthyosis, epitheliotropic T-cell lymphoma, severe atopic dermatitis with secondary infection, and dermatophytosis. The mycology culture result was negative. Biopsy under general anesthesia was conducted using a 6 mm punch biopsy machine in the ventro-lateral aspects of the thorax and lumbosacral area.

At day 90, dermatological examination was repeated with an assessment of the severity of scales, their size and distribution, as well as the intensity of pruritus. Biopsy was conducted under general anesthesia using a 6 mm punch biopsy machine in the ventro-lateral aspects of the thorax and lumbosacral area, but on the contralateral side of the previous biopsies.

### 2.2. Treatment Protocol

Treatment included oral isotretinoin at a dose of 1 mg/kg twice daily (Roaccutane, La Roche Pharma, Basel, Switzerland). Antibiotics, shampoos, emollients and moisturizing products were not used for a 3-month period.

### 2.3. Blood Analysis and Schirmer Tear Test

Hematology and serum biochemistry were conducted on admission, and after three, six and twelve months. Production of tears was evaluated after two years of isotretinoin usage.

### 2.4. Histological Examination

Biopsy samples were taken at day 0 and 90 days after starting the treatment with isotretinoin. Skin samples were fixed in 10% neutral-buffered formalin for 24 h and then embedded in paraffin wax and processed routinely.

### 2.5. Genetic Testing

Ethylenediaminetetraacetic acid (EDTA)-blood samples were sent to the laboratory (Laboklin GmbH& CoKG, Postfach1810; 97688 Bad Kissingen, Germany) for mutational analysis. DNA extraction and amplification was performed using the PCR technique described in the paper by Grall and colleagues [4]. It was previously established that wild-type genes are unaffected with no transmission of the variant. Heterozygous dogs were found to be unaffected and able to transmit the *PNPLA 1* variant to 50% of their lineage. Homozygous dogs were affected with clinical signs and it is known that they will transmit the *PNPLA 1* variant to 100% of their lineage [4,10].

Analysis confirmed that the patient was homozygous for mutation in the *PNPLA 1* gene with an autosomal-recessive trait of inheritance.

## 3. Results

History, clinical signs and histopathological changes led to the diagnosis of ARCI seen in golden retriever dogs. Genetic testing performed on a venous blood sample confirmed that the patient was homozygous for the *PNPLA 1* gene mutation.

### 3.1. Dermatological Examination and Blood Analysis

**Figure 1:**
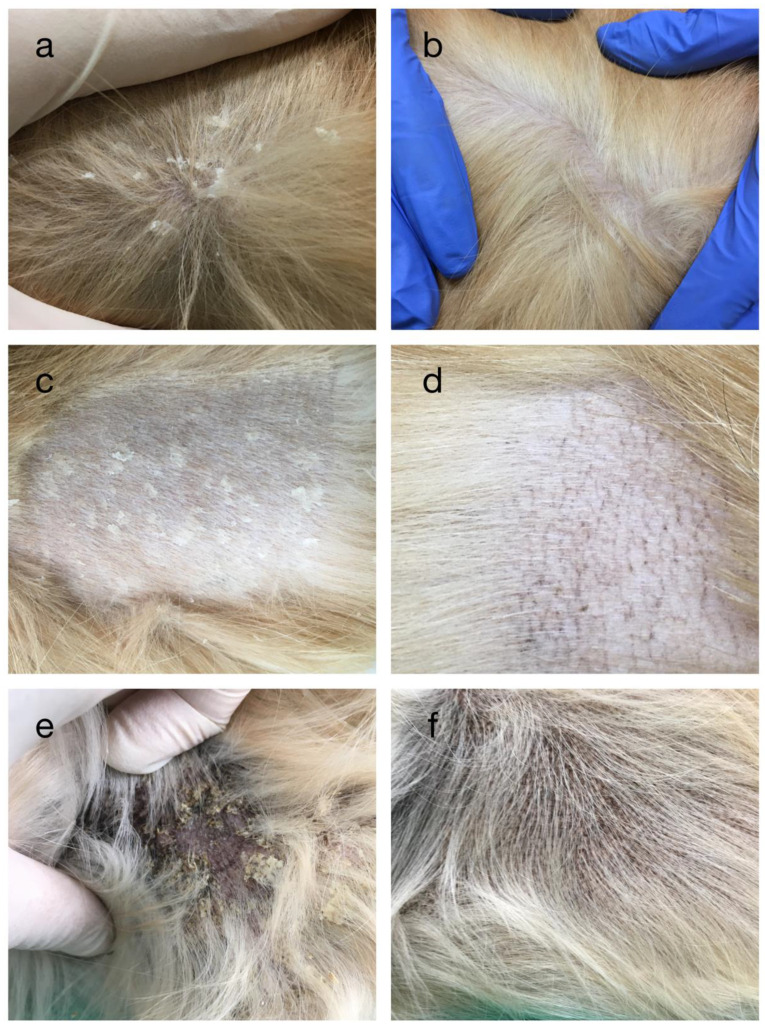
Clinical response to isotretinoin therapy in a golden retriever dog with autosomal recessive congenital ichthyosis (ARCI). Pictures on the left were obtained before and those on the right after 3 months of treatment at the same body locations but on the contralateral sides. Picture before (**a**) and after treatment (**b**) on the trunk. Picture before (**c**) and after treatment (**d**), with clipped hair for better visualization of large white scales and latticelike hyperpigmentation (**d**). Picture before (**e**) and after treatment (**f**) in axillar region showing tan-to-yellow scales with variable hyperpigmentation.

At initial presentation, severe generalized scaling was present, mostly on the dog’s trunk and with less scales on the extremities and head. Mostly medium-to-large, white scales were seen (Figure 1a). The scales were loosely adherent to the skin and some embedded hair shafts (Figure 1c). Tan-to-yellow scales were noticed in the axillary and inguinal region with moderate hyperpigmentation (Figure 1e). Periocular alopecia with moderate lichenification, ceruminous otitis and moderate pododermatitis were also noticed. Based on clinical and histopathological findings, a diagnosis of ichthyosis with a secondary atopic dermatitis was made. Pruritus was graded based on the pruritus visual analog scale (pVAS) as 5/10 [11]. The complete blood count (CBC) was within normal range while biochemical analysis revealed mild elevations in triglycerides 3.4 mmol/L (reference range 0.2–1.3) and cholesterol 8.7 mmol/L (reference range 3.5–7.1).

After 3 months of isotretinoin usage, the clinical examination showed marked improvement. Scales completely disappeared from the whole body and hyperpigmentation decreased on areas where it was originally seen (Figure 1b,d,f). When hair was clipped, latticelike hyperpigmentation was noticed (Figure 1d). Pruritus was evaluated again (pVAS 5/10). There were no side effects associated with retinoid usage. Repeated blood analysis revealed normal CBC with normal triglycerides, and increased cholesterol 10.2 (reference range 3.5–7.1).

After 3 months of 1 mg/kg twice-daily dose of isotretinoin, the dose was decreased to the lowest effective amount. The patient was clinically stable at 0.6 mg/kg twice daily for the next 18 months. Repeated CBC and biochemistry analysis results were normal in that time range. After 2 years of isotretinoin usage, therapy was stopped due to drug shortage. Scaling increased after 2–3 weeks of isotretinoin cessation. Three months later, the patient was restarted with isotretinoin therapy 0.6 mg/kg twice daily, which resulted in the disappearance of scales within 3 weeks.

During treatment with oral isotretinoin, the owner was informed about potential side effects and the development of keratoconjunctivitis sicca. After two years of treatment, a Schirmer tear test was conducted. The tear uptake was 25 mm/min in both eyes, which was considered physiological (reference range 29.3 ± 16.9 mm/min) [12].

### 3.2. Histopathological Findings

**Figure 2:**
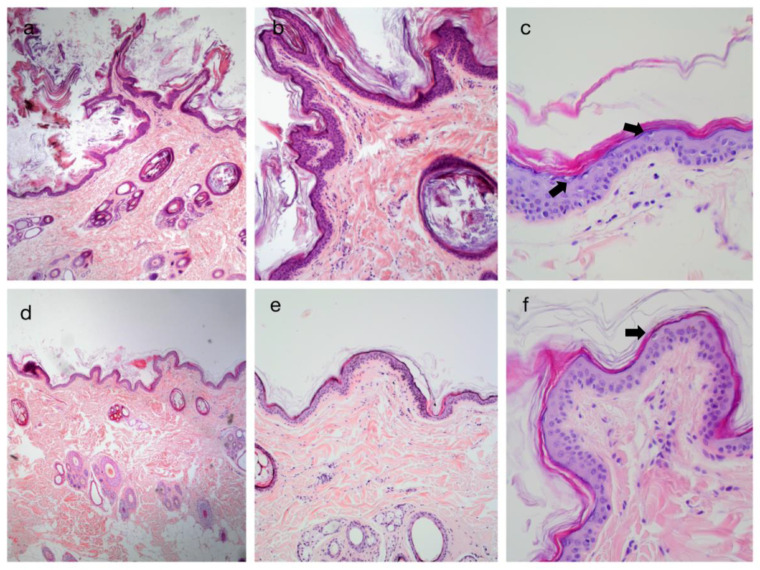
Histopathological changes in skin biopsies in a golden retriever with autosomal recessive congenital ichthyosis (ARCI), before (**a**–**c**) and after treatment (**d**–**f**) with isotretinoin. Note the diffuse and severe laminar orthokeratotic hyperkeratosis in the stratum corneum in picture (**a**) (×4), (**b**) (×10) and (**c**) (×40). After treatment with isotretinoin, keratin was not so abundant, as shown in pictures (**d**) (×4), (**e**) (×10) and (**f**) (×40), and had a more typical basket-weave appearance (pictures **e**,**f**). In picture (**c**) (×40), there is a moderate number of scattered vacuolated keratinocytes (arrows) in the epidermis, while in picture (**f**) (×40), their number is decreased.

The histological findings before and after treatment with isotretinoin are illustrated in Figure 2. The initial biopsies (Figure 2a–c) revealed moderate-to-severe diffuse lamellar orthokeratotic hyperkeratosis with multifocal black-to-brown granular depositions in the lamellar keratin. Epidermis was mostly 2–3 layers thick with multifocal mild acanthosis. The epidermis had a severe diffuse pleated appearance. Mild-to-moderate superficial-to-mid-dermal perivascular infiltration of lymphoplasmacytic cells was seen. Moderate-to-severe follicular lamellar hyperkeratosis was noticed in the superficial dermis. A moderate number of vacuolated keratinocytes were scattered in the epidermis (Figure 2c).

Histological findings after treatment with isotretinoin (Figure 2d–f) revealed marked improvements. Mild-to-moderate multifocal orthokeratotic hyperkeratosis was seen, but keratin was more organized in a basket-weave appearance. The epidermis was mildly acanthotic with a mild-to-moderate multifocal pleated appearance. Mild superficial perivascular infiltration of lymphoplasmacytic cells was noticed. Mild superficial follicular hyperkeratosis was seen. The number of vacuolated keratinocytes decreased.

## 4. Discussion

Our clinical and histopathological findings indicate that treatment with oral isotretinoin was effective in improving ichthyosis without any side-effects. To the best of the authors’ knowledge, this is the first clinical and histological investigation of oral isotretinoin in ichthyoses in canine patients.

The first clinical signs of ichthyosis can be seen within the first 3 weeks of life but sometimes it can take up to 3 years to be clearly manifested [3]. Scales in golden retrievers with ARCI are usually white-to-grey, loosely adherent and mostly present on the trunk with sparing of the head and extremities. Clipping off the hair allows better visualization of large adherent, fine, polygonal scales [2,13]. Sparsely haired areas, such as the abdomen and axillae, are hyperpigmented and have a rough, sandpaper-like appearance [3]. Rarely, ceruminous otitis and hyperkeratosis of the paw pads can be seen [3].

Histological changes of ichthyotic skin show laminated or compact orthokeratotic hyperkeratosis with frequent melanic pigmentation. The basal membrane is often undulated and has a “pleated” appearance, which explains the rough, sand-paper-like appearance of the skin [3]. When compared to histological pictures after treatment with isotretinoin, there is a marked improvement in lamellar orthokeratotic hyperkeratosis, which has a more normal, basket-weave-like appearance. In addition, histologic improvement is seen clinically as a loss of white, loosely adherent scales.

Retinoids are structurally and functionally related to vitamin A [14] with proven functions in the skin based on the modulation of epidermal maturation, as well as keratinocyte differentiation, apoptosis, immune function and carcinogenesis [15,16]. Moreover, isotretinoin was found to induce apoptosis and cell cycle arrest in human sebocytes [17]. As a result, retinoids decrease thickening and scaling of the skin, decrease erythema [14,18] and facilitate wound healing [19]. Systemic retinoids have been successfully used for ARCI in human medicine [20], but until now they have not been used in veterinary medicine for ichthyoses.

Isotretinoin was developed as a synthetic retinoid, but then it was discovered that this 13-*cis* isomer of naturally occurring tretinoin (*trans*-retinoic acid) was present in cells as a naturally occurring metabolite [21]. Isotretinoin has a short half-life (~1 h) [22]; however, clearance from the organism can take several months [21]. The recommendation is to use the lowest effective dose to decrease side effects [21]. Isotretinoin has a low teratogenic risk but is advised not to be used in pregnant individuals. Monitoring for complete blood count with liver function tests is recommended. The most commonly changed parameters are triglycerides and cholesterol. In human medicine, acute toxicities connected with retinoid usage are cheilitis, xerosis, dry nose, eye irritation and hair loss. Chronic usage of retinoids in human medicine is mostly manifested in the skeletal system (enthesopathy, hyperostosis or spurs along the spine) [16,21]. In human and veterinary medicine, retinoids are known to alter the tear composition by decreasing the lipid component in the tears. As a result, increased tear evaporation leads to keratoconjunctivitis sicca [14,23].

Clinical improvement in the golden retriever was noticed within 3 weeks of treatment, which is compatible with treatment of human ARCIs where thickness and scaling decreases in two weeks from staring the therapy [21]. After discontinuation of isotretinoin in humans, symptoms usually reoccur, which was also seen in our case. Transient elevations of cholesterol and triglycerides were noticed, but were also present before the therapy with isotretinoin. After several months of therapy with isotretinoin and the decreasing of the dose to the lowest effective amount, all biochemical abnormalities resolved and were within reference range. Ichthyosis causes a major epidermal barrier defect and is not so rare that atopic dermatitis (AD) is secondarily manifested. In human patients, certain populations with ichthyosis vulgaris are at increased risk for developing AD due to barrier defects [24]. Golden retrievers are predisposed to developing AD, with their heritability estimated to be 0.47 [25]. Golden retrievers are also predisposed for ARCI, though the heritability is not known. However, at the present time, no link has been established between the two diseases. Further studies are needed to compare incidence of AD in dogs with or without ARCI in golden retrievers. Isotretinoin did not have any effect on pruritus in our case. The most probable explanation lies in the fact that pruritus develops due to sensitization to allergens as a consequence of a barrier defect and not as a primary symptom of ARCI. As ARCI is a congenital disease, the treatment with isotretinoin could be recommended throughout life; however, long-term side effects have not been deeply investigated and larger studies are needed. Clinicians need to estimate the benefit–risk balance before prescribing an oral retinoid for long-term use. It is advised to monitor liver enzymes, lipids and tear production during isotretinoin treatment.

We could say that ARCI is a cosmetic disease that needs no systemic treatment; however, the subject of our case had a history of constant antibiotic usage due to severe secondary bacterial and fungal infections that were almost impossible to control. After treatment with isotretinoin, the secondary infections almost disappeared completely and the symptoms of AD were easily controlled.

Further controlled studies on larger samples are needed to confirm the beneficial impact of isotretinoin in dogs with ARCI and the general effects of the long-term usage of isotretinoin in this species.

## 5. Conclusions

Clinical and histopathological findings indicate that treatment with oral isotretinoin was effective in improving symptoms of autosomal recessive congenital ichthyosis in a golden retriever. Isotretinoin appeared safe and well tolerated, with no observed side-effects.

## Data Availability

The data presented in this study are available in the manuscript.
